# Molecular Insights of 1,2,3,4-tetrahydropyrimido[1,2-a]benzimidazole as CRF-1 Receptor Antagonists: Combined QSAR, Glide Docking, Molecular Dynamics, and *In-silico* ADME Studies 

**DOI:** 10.22037/ijpr.2020.113746.14464

**Published:** 2021

**Authors:** Sunil Kumar, Neeraj Kumar, Chandra Shekhar Sharma, Shashank Shekher Mishra

**Affiliations:** *Department of Pharmaceutical Chemistry, Bhupal Nobles’ College of Pharmacy, Bhupal Nobles’ University, Udaipur-313001, India.*

**Keywords:** Stress-dependent disorders, Corticotropin-Releasing Factor-1, 1, 2, 3, 4-tetrahydropyrimido[1, 2-a]benzimidazole derivatives, Molecular docking, Molecular dynamics simulations, QSAR

## Abstract

Stress-dependent disorders cause severe harm to human health and trigger the risk of neurodegenerative disorder. Corticotropin-releasing factor-1 receptor was found to be a potent drug target.We evaluate the essential structural residues for pharmacophore identification through 2D and 3D QSAR analysis and identify the binding residues for a possible mechanism of CRF-1 binding with 1,2,3,4-tetrahydropyrimido[1,2-a]benzimidazole derivatives through molecular docking and molecular dynamics simulations. The best 2D QSAR model was obtained through the MLR method with an r^2^ value of 0.8039 and a q^2^ value of 0.6311. Also,a 3D QSAR model was generated through the KNN MFA method with a q^2^ value of 0.6013 and a q^2^_se value of 0.3167. Further, docking analysis revealed that residue Glu196 and Lys334 were involved in hydrogen bonding and Trp9 in Π- Π stacking. Simulation analysis proves that target protein interactions with ligands were stable, and changes were acceptable for small and globular proteins. Compound **B18**, a benzimidazole derivative, has an excellent binding affinity towards CRF-1 protein compared to reference molecules; hence, this compound could be a potential drug candidate for stress-dependent disorders. Based on findings, 1,2,3,4-tetrahydropyrimido[1,2-a]benzimidazole derivatives could be a novel class of corticotropin-releasing factor 1 receptor antagonists for stress-related disorders. All benzimidazole derivatives were found to be within the acceptable range of physicochemical properties. Hence, these observations could provide valuable information for the design and development of novel and potent CRF-1 receptor antagonists.

## Introduction

Depression, anxiety and stress are the most prominent psychiatric disorders and play an important role in the onset of neurodegenerative disorders (1). Stress is a life-saving mechanism that has been figured and refined throughout evolution, and anxiety is a general response to stress (2, 3). Exposure to stress leads to a decrease in cognitive performance and anincrease in memory consolidation and can contribute to Alzheimer’s disease (4-7). In stress-related disorders, the stress response is an extremely arranged mechanism whereby the body expeditiously activates the hypothalamic-pituitary-adrenocortical (HPA) axis and autonomic nervous system (8, 9). This activation is responsible for releasing an enormous number of neuropeptides, neurotransmitters and hormones to restore homeostasis as adaptive reactions (9). 

Corticotropin-releasing factor (CRF) is a principal mediator of the stress response on the HPA axis due to its ability to integrate physiological responses to react against a stressor (10). CRF is a 41-amino acid-containing polypeptide secreted at the onset of stress in the paraventricular nucleus of the hypothalamus and implicated in depression and anxiety disorders (11-14). CRF regulates the HPA axis at the pituitary level and triggers stress-related events like secretion of the corticosteroids in the adrenal cortex (14). Various research investigations prove that elevated CRF levels were found in behavioral- and stress-related physiological disorders (13-15). The actions of CRF are mediated through two types of G-protein coupled receptors, CRF-1 and CRF-2 and CRF-1 receptor is mainly distributed throughout the central and peripheral nervous system (16). Many studies have been conducted to discern the roles of CRF-1 and CRF-2 receptors in stress-related physiological and behavioral processes to gain insight into anxiety and major depressive disorders. CRF-1 binds with CRF receptors with high affinity and mediates the effects of CRF. It has been proved that CRF-1 deficient mice show reduced anxiety-related behavior, and overexpression of CRF in transgenic mice show increased anxiety-related disorders (17-20). In keeping with this, the concept is dawning that CRF, its congeners and their receptors form an intricate network in the brain that potentially provides various targets for drug intervention. These findings lead that CRF-1 is an attractive target for drug development programs for stress-related disorders. 

There are various CRF-1 receptor antagonists developed which still, no one can become potential drug due to various factors, including adverse side effects, tolerance, long latency of clinical effect and additive potential. Hence, development of potential CRF-1 receptor antagonists is quite essential to overcome stress related disorders. 

Computer-aided drug design (CADD) is a smart way to design and develop novel molecules. This approach reduces the cost of the drug discovery program and minimizes the chance of failures in the final step (21, 22). Quantitative structure-activity relationship study is a credible strategy in drug discovery which defines the insights of variation in chemical structure toward the biological activity for differentiating drug-like from nondrug-like molecules (23).

3D quantitative structure-activity relationships (3D QSAR) and pharmacophore modeling are the most important and widely used tools in ligand-based drug design. In this investigation, the ligand-based drug design approach was applied for pharmacophore identification and binding pattern recognition. We performed QSAR modeling of selected benzimidazole derivatives to identify essential pharmacophoric features and performed docking and dynamics simulation analysis to predict binding mechanism. In addition, we had executed physicochemical parameters calculations for predicting the drug-like ability. We hope this information could be used to facilitate thedevelopment and design of novel CRF-1antagonists.

## Experimental

The 1,2,3,4-tetrahydropyrimido[1,2-a]benzimidazole derivatives with the antagonistic activity of the CRF-1 receptor (24) as selected for the computational study (Supplementary File, Table S1). The biological activity of derivatives was IC_50_ in the nanomolar unit which was expressed in molar scale and converted into a negative logarithmic value (pIC_50_).

*Pharmacophore modeling*

To identify the pharmacophore, we performed 2D and 3D QSAR analysis through VLife Molecular Design Suite (VLife MDS; Supplied by VLife Science Technologies, Pune, India). 

For 2D QSAR, physicochemical and alignment independent 2D descriptors were calculated in the 2D-QSAR module with the removal of invariable descriptors.Further, the data set was divided into test and training sets. Test and training data set selection was validated by unicolumn statistics. Multiple linear regression (MLR) method coupled with the stepwise,forward and backward variable selection models was applied for generating the best 2D QSAR model with default settings. 

For 3D QSAR, the k-nearest neighbor molecular field analysis (kNN MFA) method was applied with a common rectangular grid generation to align a set of molecules. The steric and electrostatic field descriptors were calculated with distance-dependent dielectric function. The carbon atom was selected in probe settings with charge 1.0, electrostatic and steric cutoffs selected as 10.0 and 30.0 kcal/mol, respectively. In addition, data sets were divided into training and test sets. The 3D QSAR equation was generated by the kNN MFA model coupled with the stepwise, forward and backward variable selection models. 

*Molecular docking*

For evaluation of binding pattern with CRF-1 protein, molecular docking analysis was performed (25). The crystal structure of the extracellular domain of human corticotropin-releasing factor receptor type 1 (CRFR1) in complex with CRF (PDB ID: 3EHT) was retrieved from Protein Data Bank (PDB) (26). The co-crystallized structure was prepared by the protein preparation tool where missing loops, hydrogens and atoms were added to the structure to make them ready for the next step. In the next step, restrained minimization was done to remove steric clashes of the atoms and hydrogen bond assignment using Propka. The reported CRF-1 antagonists such as Emicerfont, Verucerfont and Pexacerfont were selected as references, and further ligands were prepared using the Ligprep tool. All possible tautomers and low energy ring conformation states were generated for each ligand. Glide-grid was generated as 20 Å size into the ligand binding region by removing the co-crystallized ligand CRF. Docking was executed in extra precision mode with the selection of post docking minimization (25).

*Binding energy calculation*

The data set was carried out for binding free energy calculations by using the Prime/MMGBSA tool of the maestro. Local optimization feature was used to minimize the docked poses in prime, where the energies of the complexes were computed using the OPLS-AA 2005 force field and generalized-Born/surface area (GB/SA) continuum solvent model (27). The binding free energy was computed using the below Equation:

∆G_bind_= ∆E+ ∆G_solv_+ ∆G_SA_

Where, ∆G_bind _is the binding free energy, ΔE, ∆G_solv_ and ∆G_SA_ are considered as the minimized energy, solvation free energy and surface free energy of the complexes. 

*Molecular dynamics simulations*

Molecular dynamics simulation analysis was performed through the Desmond tool of Schrodinger for the protein-ligand complex. Among all evaluated compounds, the top one compound was selected for simulations based on binding energy, hydrogen bonds and docking scores. In the present work, we selected compound **B18 **for MD simulation studies. Prior to simulation, a solvated system was generated by selecting POPC as membrane, SPC as a solvent model with a 10.0 Å box size. The salt concentration was maintained as 0.15 M of Na^+^ and Cl^-^ ions to achieve physiological conditions.

Furthermore, the energy of the solvated complex was minimized by a maximum of 2000 iterations. The processed complex was retrieved from the workspace to the molecular dynamics window, and the NPT ensemble was set at 1.01325 bar pressure and 300.0 K. Additionally, after model system relaxation, a 20ns simulation was performed, and a 4.8 ps time interval was set for trajectory recording. In addition, conformational behavior and complex stability were analyzed through ligand-protein RMSD, RMSF, protein-ligand contacts charts. 

*Physicochemical parameters calculation*

Physicochemical parameters evaluation is essential to design an ideal drug. Hence, we evaluated all compounds for physicochemical parameters calculations using the QikProp tool of the maestro. 

## Results and Discussion

*Validation of 2D and 3D QSAR model*

*For 2D QSAR*

The substituted benzimidazole derivatives were selected and divided into training and test sets with16 and 4 compounds, respectively. The data set selection was validated through uni-column statistics (Table 1).

The maximum of the test set was found to be less than the maximum of the training set and the minimum of the test set was greater than the minimum of the training set. Hence, the test set was found to be interpolative within the maximum-minimum ranges of the training set along with the standard deviation of statistics. This validated data set was applied to generate a robust 2D QSAR model via different statistical methods. The best significant QSAR model was obtained through multiple linear regression (MLR) coupled with stepwise, forward and backward methods. The model is given in Equation 1:

pIC_50_ = -1.04 (T_N_O_6) -0.20 (T_2_N_6) 0.25 (chi1) + 5.22              Equation 1.

Where T_N_O_6, T_2_N_6 and chi1 are the descriptors indicating their position along with their respective coefficients and regression constant is the last numerical term in this equation. 

The other statistical parameters used to evaluate the quality and stability of the model are given in Table 2. 

The squared correlation coefficient explains the quality of fit by 80% of the total variance in the training set. A high r^2^ and low standard error r^2^ (r^2^_se) value of 0.8039 and 0.2492 denotes the accuracy of the obtained model. It also has an internal (q^2^) and external (pred_r^2^) predictive ability of ~63% and ~49%, respectively. These parameters indicate that obtained model indicated an excellent correlation between activity and physicochemical descriptors. Thus obtained MLR obtained model is robust. 

**Figure 1:**
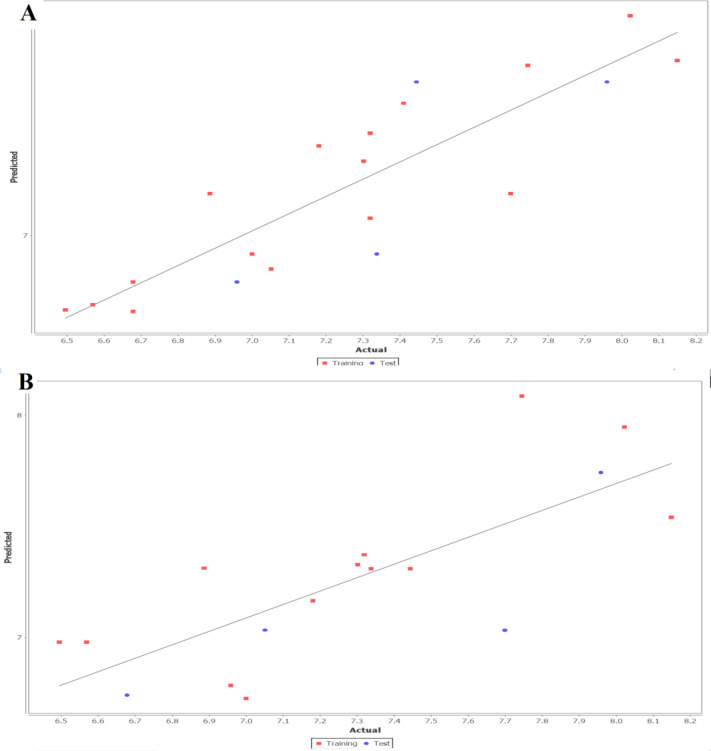
Linear fitness plot illustrating the correlation of predicted versus actual activity for training and test set for (A) 2D QSAR and (B) 3D QSAR model

The fitness plot describes an idea about how well the model was trained and how well it predicts the activity of the external test set (Figure 1). In this plot, points were near to the regression line showing true prediction of training set activity and external test set. The predicted and residual activity of data sets was given in Table S1 of supplementary data.

*For 3D QSAR*

The robust 3D QSAR analysis was carried out by applying the k-nearest neighbor molecular field analysis (kNNMFA) method coupled with the stepwise, forward and backward methods. All compounds were again divided into training and test data sets, including 13 and 7 compounds, respectively. The data selection was validated through uni-column statistics (Table 1) and found to be interpolative within the maximum-minimum ranges of training set along with standard deviation of statistics. 

Furthermore, the kNN MFA model coupled with the stepwise, forward and backward methods was applied after a common rectangular grid generation around the co-crystallized compounds. The descriptors, along with other statistically significant parameters, are given in Table 2. According to statistical results, the obtained model was found to comparatively better in terms of the internal (q^2^ = 0.6013) as well as the external (pred_ r^2^= 0.5338) model validation and accurately predicted the activity ~60% and ~53% for the training and test set respectively. The obtained model describes that electrostatic interactions (E_1108) play a significant role in determining CRF-1 antagonistic activity. The difference between observed vs. predictive activity in distance point terms (Fitness plot) is shown in Figure 1. The predicted and residual activity of data sets was given in Table S1 of supplementary data.

*Interpretation of pharmacophore model*

*Contribution of 2D parameters*

**Figure 2:**
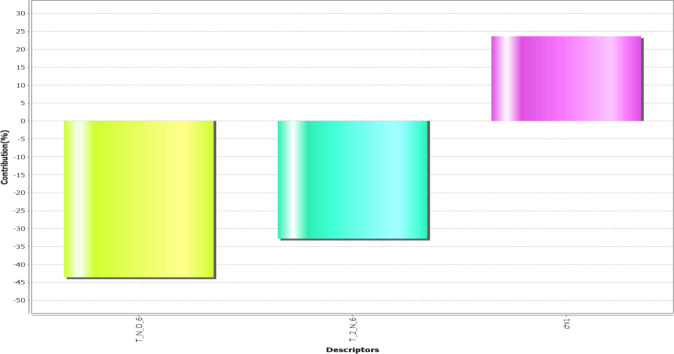
2D parameters contribution plot for training and test set

The obtained model revealed that the descriptors T_N_O_6, T_2_N_6, and chi1 play the most prominent role in predicting activity explaining the correlation with standard to the variation in different substitution sites (Figure 2).

T_N_O_6: This alignment-independent descriptor is the count of number of nitrogen atoms (single, double or triple bonded) separated from oxygen atom by 6 bond distance in a molecule. This descriptor shows a negative contribution in terms of percentage is 45%.

T_2_N_6: This alignment-independent descriptor is the count of number of double-bonded atoms (*i.e*., any double-bonded atom, T_2) separated from the nitrogen atom by 6 bonds. This descriptor also shows a negative contribution in terms of percentage is 34%.

chi1: This physicochemical descriptor signifies a retention index (first-order) derived directly from gradient retention times. It shows positive contribution in terms of percentage is 25%.

*3D QSAR and pharmacophore modeling*

**Figure 3:**
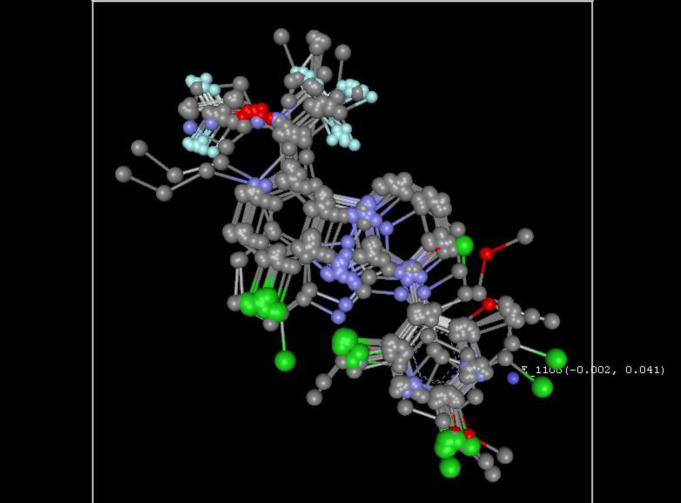
3D plot of the common rectangular grid around the nucleus through the kNN-MFA model

The calculated field descriptors were utilized for the evaluation of the activity of the compounds. The steric and electrostatic energies are computed at the lattice points of the grid using a methyl probe of charge +1. These interaction energy values at the grid points are considered for relationship generation using the kNN method and utilized as descriptors for obtaining distances within this method. Figure 3 shows the relative position and ranges of the corresponding important electrostatic/steric fields in the model provides the following guidelines for the design of a new molecule. 

For the electrostatic field, the negative range indicates that negative electrostatic potential is favorable for increased activity. Hence, a more electronegative substituent group is preferred in that region, and a positive range exhibited that positive electrostatic potential is favorable for an increase in the activity. So, a less electronegative substituent group is preferred in that region. Consequently, developed kNN-MFA model, one electrostatic fields range (E_1108: -0.0015 to 0.0406) shows the range is more towards the negative side, and hence, increasing electronegativity of the substituent group is favourable at the 1,2,3,4-tetrahydropyrimido-[1,2-*a*]benzimidazole core.

Therefore, in the context of 2D and 3D QSAR, the pharmacophore responsible for CRF-1 antagonistic activity was investigated. The compound **B3 **(IC_50_=11 nM) with tetrahydropyrimidobenzimidazole core was potent as the compound **B2 **(IC_50_=18 nM) of benzimidazole with a trisubstituted phenyl group. The presence of an electronegative atom like Cl atom at the 9-position of compound **B7 **(IC_50_=7.1 nM) increases the binding activity. Hence, it was a promising lead for further development work. Removal of diethylamino group on the 6-position through hydroxyl group (IC_50_=89 nM), cyano group (IC_50_=66 nM), and methoxy group (IC_50_=20 nM) maintained the activity and reducing the lipophilicity. Compound **B18** has lower binding activity but improved metabolic stability due to the presence of fluorine, a more electronegative atom. Substitution with pyrimidine ring improves solubility by decreasing the lipophilicity of the compound. Thus, the presence of more electronegative atoms at the 6-position improves the binding activity and metabolic stability. 

*Docking analysis*

To explore the scope of work, we performed molecular docking analysis for selected data sets. The co-crystallized protein (PDB ID: 3EHT) has been selected, and the grid was generated around the ligand prior to XP docking. The docking results are described in Table 3.

**Figure 4:**
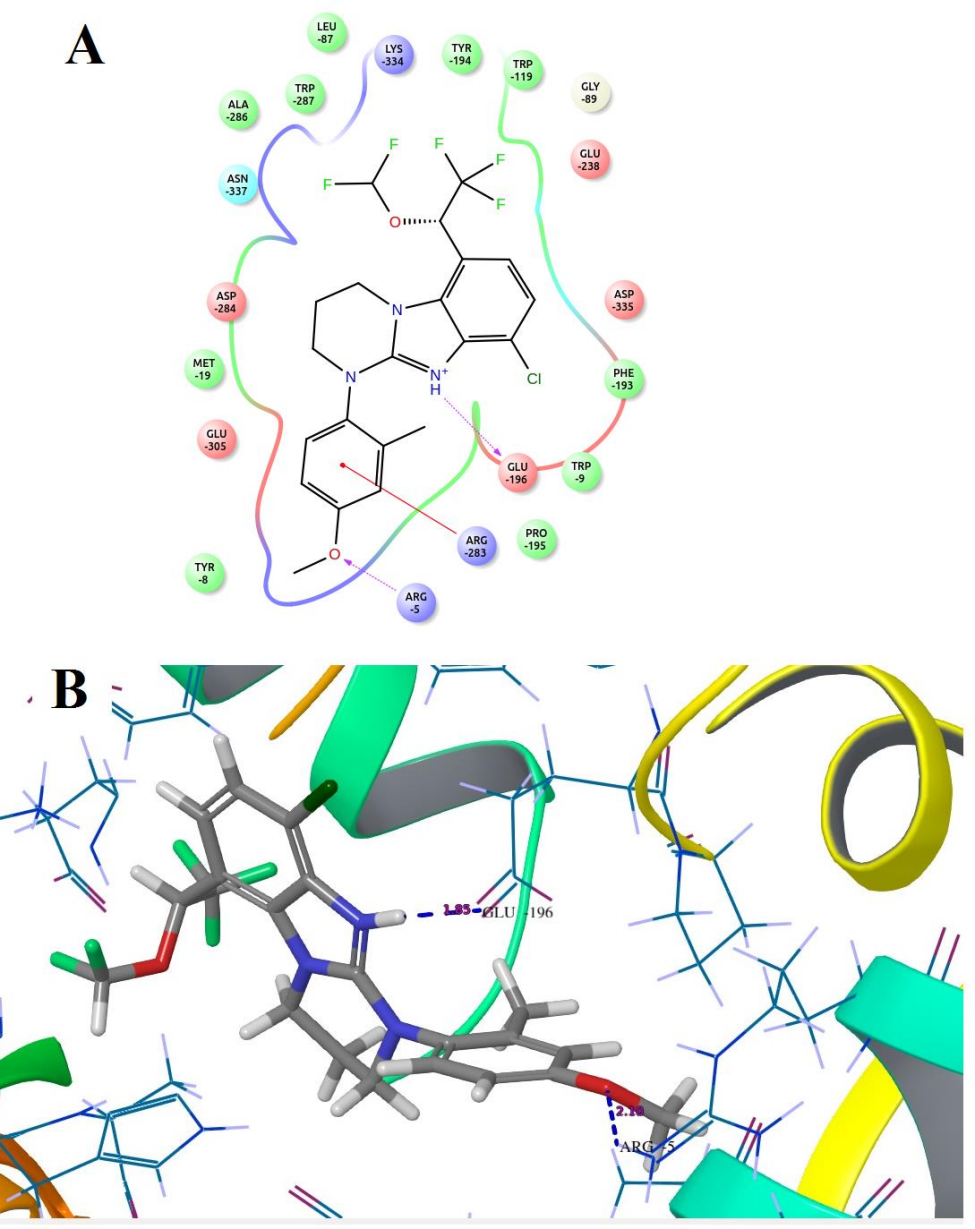
Docked compound **B18** with the target protein **(A)** Ligand interaction diagram **(B) **3D diagram

Compound **B18 **(9-chloro-6-(1-(difluoromethoxy)-2,2,2-trifluoroethyl)-1-(4-methoxy-2-methylphenyl)-1,2,3,4-tetrahydrobenzo[4,5]imidazo[1,2-a]pyrimidine) has the highest docking score -8.920 and shows 2 hydrogen bonds with residue Arg5 and Glu196. It also shows one Π-cation interaction with Arg283. In reference compounds, **CP-316,11 **has the docking score -6.889 and shows Π-Π stacking with Tyr194, Trp287 and Salt bridge with Glu305, Glu196 residues. The ligand interaction and 3D diagram of compound **B18 **are given in Figure 4.

**Figure 5:**
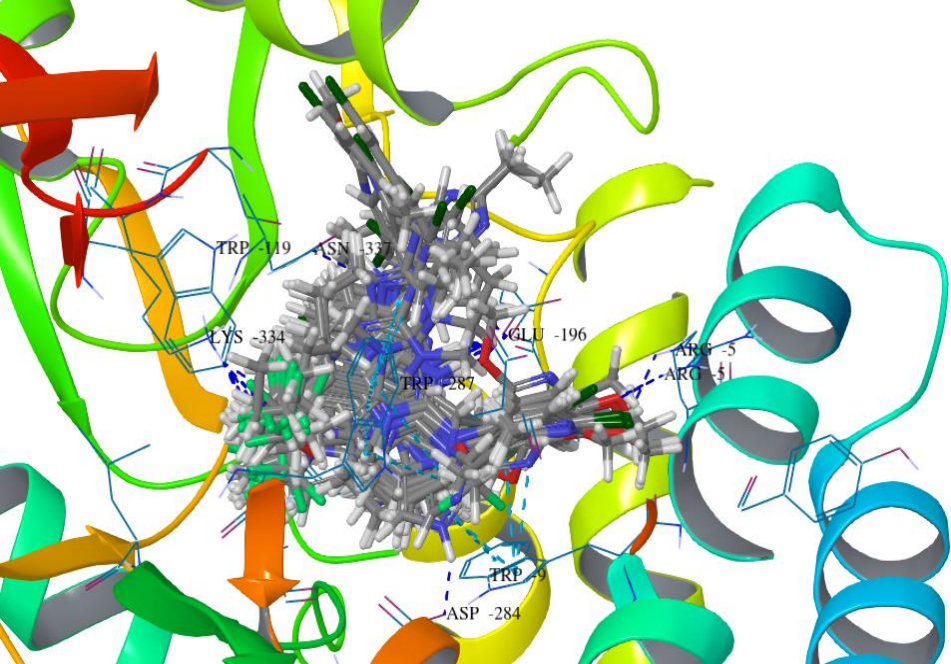
The binding pattern of superimposed docked compounds in the binding pocket of 3EHT protein showing H-bond interactions

The nitrogen of the benzimidazole nucleus involves in hydrogen bond formation for better binding and benzene ring forms Π-cation interaction with Arg283 residue. The most interesting fact is that all benzimidazole compounds show hydrogen bonding (Glu196, Lys334, Arg5, Arg283, Glu238, Asp284 and Asn199). Overall, compound **B18 **has the highest binding affinity towards the binding pocket of 3EHT protein. The hydrophobic interactions enhance the binding affinity between drug-protein interfaces; therefore, incorporation of hydrogen bonding can be helpful to optimize the binding affinity due to hydrophobic interactions (28). Hence, comparison with reference molecules suggests that all benzimidazole compounds have an excellent binding affinity towards the same binding pocket of protein (Figure 5). The presence of OCH_3_, Cl or polar substituents at 1-position enhances the binding affinity. 

Docking results revealed that the binding pocket consists of hydrophobic (Pro195, Phe193, Trp119, Leu87, Ala286, Met19, Tyr8, Tyr139 and Tyr194), and hydrogen bonding (Arg283, Glu196, Lys334, Arg5, Glu238, Asp284 and Asn199) amino acid residues. Other amino acid residues are also involved in Π- Π stacking, Π-cat and salt bridge formation. 

*Binding energy calculation*

The binding energy of the selected data sets was calculated and given in Table S2 of supplementary data. Compound **B7 **has the highest binding energy -107.147 kcal/mol.In a reference molecule, emicerfont has the highest binding energy -62.828 kcal/mol. Compound **B18 **has the dG bind energy -89.375 kcal/mol which is higher among all reference compounds. 

*MD simulation analysis*

**Figure 6:**
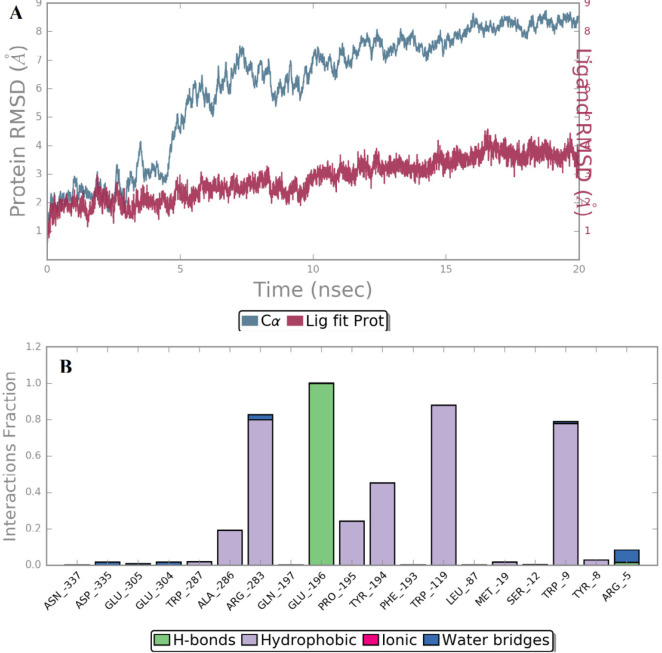
(A) Timeline representation of RMSD profile of Cα of 3EHT with respect to its coordinates and (B) Ligand-protein contacts of compound **B18**

The independent 20 ns atomistic MD simulation was performed to obtain insights into the dynamical behavior of the highest potent compound **B18 **at the trans-membrane pocket of CRF-1 protein. The system reached convergence through 20 ns simulation which is enough to determine the complex stability more precisely. The structural stability of protein-ligand complex was assessed by root mean square deviation (RMSD) which denotes the measure in the average change in displacement of a selection of atoms for a particular frame with respect to a reference frame (25). The RMSD of Cα of the simulated trajectories is shown in Figure 6A. The RMSD value of Cα was found to increase up to a value of 6.0 Å with respect to its starting coordinatefor the first 10 ns and stabilize around an average value of 8.0 Å for the rest of the MD trajectories which indicate a significant change in protein backbone compared to crystal structure. 

In addition, the root mean square fluctuation (RMSF) of the sidechain of 3EHT is found to be 5.25 Å which indicates a lower degree of flexibility in that region. It suggests lower conformational changes in C-terminal and N-terminal residues. It is clear that ligand’s movement was stable during the simulation. The observed Ligand-protein contacts were depicted in Figure 6B. Compound **B18 **(9-chloro-6-(1-(difluoromethoxy)-2,2,2-trifluoroethyl)-1-(4-methoxy-2-methylphenyl)-1,2,3,4-etrahydrobenzo[4,5]imidazo[1,2-a]pyrimidine) shows hydrogen bonding with Glu196, water bridge with Arg5, Arg283, Glu304 and Asp335. The residues involved in hydrophobic interactions were Trp287, Ala286, Arg283, Pro195, Tyr194, Trp119, Met19, Trp9 and Tyr8. It is evident from the above discussions that hydrophobic and hydrogen bonding interactions are a major contributing factor for stabilizing compound **B18 **at the trans-membrane pocket of 3EHT which is in accordance with docking results. 

The ligand torsions plot summarizes the conformational evolution of every rotatable bond (RB) in the ligand throughout the simulation trajectory. Each rotatable bond torsion is accompanied by a dial plot and bar plots of the same color. Dial (or radial) plots denotes the probability density of the torsion throughout the simulation. 

*Physicochemical parameters calculation*

In the drug discovery process, the ideal drug candidate under consideration needs to possess high efficacy as well as excellent pharmacokinetic profiles to confirm their action and potency. The acceptable ranges of crucial pharmacokinetic properties and the predicted properties of all selected compounds are listed in Table S3 of supplementary file. All evaluated physicochemical properties were found to be in their permissible range and therefore confirming their drug-like abilities. 

**Figure 7:**
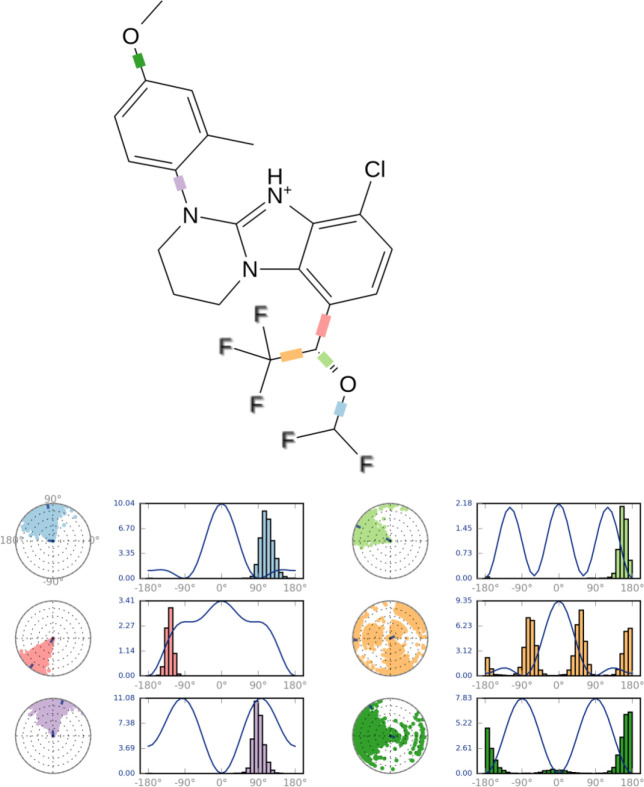
Snapshot of ligand torsion profile of compound **B18**

**Table 1:** Uni-column statistics results for 2D and 3D QSAR analysis

		**Average**	**Max**	**Min**	**StdDev**	**Sum**
2D QSAR	Activity (Training set)	7.2188	8.1490	6.4950	0.5033	115.5000
	Activity (Test set)	7.4247	7.9590	6.9590	0.4125	29.6990
3D QSAR	Activity (Training set)	7.2619	8.1487	6.4949	0.5016	94.4051
	Activity (Test set)	7.3465	7.9586	6.6778	0.5870	29.3860

**Table 2:** Statistical results of the 2D and 3D QSAR models for the CRF-1 dataset

**Statistical parameters**	**2D QSAR**	**3D QSAR**
N (training/test)	16	13
Degree of freedom	12	11
r^2^ (squared correlation coefficient)	0.8039	-
q^2^ (internal predictive ability)	0.6311	0.6013
F test	16.3927	-
r^2^_se (correlation coefficient standard error)	0.2492	-
q^2^_se (internal predictive ability standard error)	0.3418	0.3167
pred_r^2^(external predictive ability)	0.4918	0.5338
pred_r^2^se (standard error)	0.3394	0.4063
k Nearest Neighbour	-	02
Contributing descriptors	T_N_O_6, T_2_N_6, chi1	E_1108

**Table 3:** Glide Gscore, hydrogen bond and other main interactions involved in ligand-receptor stabilizing and reference molecules with the CRF-1 target protein

**Compound** **code**	**Glide Gscore**	**No. of H bond**	**Residues involved in H bond**	**Other interactions**
**B1**	-7.159	02	Glu196	-
**B2**	-7.487	02	Glu196	-
**B3**	-6.547	01	Glu196	-
**B4**	-7.395	02	Arg283, Glu196	-
**B5**	-7.532	01	Glu196	-
**B6**	-8.113	01	Glu196	Π-cat with Trp9
**B7**	-6.194	01	Glu196	-
**B8**	-7.908	03	Glu196, Lys334, Glu238	Π-cat with Arg283
**B9**	-7.346	02	Glu196, Lys334	-
**B10**	-7.687	01	Glu196	-
**B11**	-8.664	01	Asp284	Π- Π stacking with Trp9, Arg283 and Salt bridge with Asp335
**B12**	-8.664	01	Asp284	Π- Π stacking with Trp9, Arg283 and Salt bridge with Asp335
**B13**	-7.967	02	Glu196, Lys334	-
**B14**	-8.041	03	Arg5, Glu196, Lys334	Π-cat with Arg283
**B15**	-8.348	02	Arg5, Glu196	-
**B16**	-7.602	02	Glu196, Asn199	-
**B17**	-7.355	02	Glu196, Lys334	Π-cat with Arg283 and Π- Π stacking with Trp9
**B18**	-8.920	02	Arg5, Glu196	Π-cat with Arg283
**B19**	-7.533	02	Glu196, Lys334	-
**B20**	-8.060	02	Glu196, Lys334	-
**CP-316,11**	-6.889	01	Asn337	Π- Π stacking with Tyr194, Trp287 and Salt bridge with Glu305, Glu196
**Emicerfont**	-6.406	02	Glu238, Lys334	-
**Verucerfont**	-5.834	01	Asp335	-
**Pexacerfont**	-3.417	01	Arg5	Π- Π stacking with Trp287 and Salt bridge with Arg283

## Conclusion

In the present investigation, pharmacophore modeling, molecular docking, MM/GBSA, ADME, and molecular dynamics simulation studies were performed to identify structural determinants responsible for CRF-1 antagonism. The obtained 2D QSAR model gave an r^2^ value of 0.8039 and a q^2^ value of 0.6311, indicating excellent consistency and robustness of the model. Generated 3D QSAR model suggests the E_1108 descriptor plays a significant role with a q^2 ^value of 0.6013. Statistical parameters results prove the robustness of the obtained model. The kNN-MFA model 3D plot revealed the electrostatic field descriptor position contributing to increase the activity. The presence of more electronegative atoms at 6-position enhances metabolic stability and activity.

Further, molecular docking analysis predicted the binding mode of the selected antagonist at the binding site of CRF-1. The decisiveness of the docking study was confirmed by a low RMSD value of 6.0 Å between the co-crystal and docked ligand. Docking results suggest that the polar region forms the hydrogen bonding network with Arg283, Glu196, Lys334, Arg5, Glu238, Asp284 and Asn199 amino acid residues. MD simulation revealed that no ionic interactions between amino acid residue and compound** B18 **were reported. Molecular docking results revealed that compound **B18 **showed the highest binding affinity towards the binding pocket of 3EHT protein compared to all reference molecules. MD simulation results indicate that compound **B18 **(9-chloro-6-(1-(difluoromethoxy)-2,2,2-trifluoroethyl)-1-(4-methoxy-2-methylphenyl)-1,2,3,4-etrahydrobenzo[4,5]imidazo[1,2-a]pyrimidine) movement was stable during the simulation, and major interaction was found with Glu196 in terms of hydrogen bonding. Hence, this work can provide the lead for benzimidazole-based drug discovery program for stress-related disorders. Various physicochemical and relative binding energy parameters were also calculated for all compounds. Therefore, substituted 1,2,3,4-tetrahydropyrimido[1,2-a]benzimidazole compounds could be a selective, efficacious and potent for treating anxiety, depression and stress associated disorders. The outcomes of the present work provide insightful information regarding the design and development of novel CRF-1 antagonists to treat stress dependent disorders. 

## Supplementary Materials


