# Supplementary material for: Molecular Insights of 1,2,3,4-tetrahydropyrimido[1,2-a]benzimidazole as CRF-1 Receptor Antagonists: Combined QSAR, Glide Docking, Molecular Dynamics, and In-silico ADME Studies
Source: Iran J Pharm Res. 2021 Spring;20(2):22–34. doi: 10.22037/ijpr.2020.113746.14464 (PMC8457742; doi:10.22037/ijpr.2020.113746.14464)
Supplement: Supplementary file 1 [file ijpr-20-22-s001.pdf]

Supplementary Materials for

Molecular Insights of 1,2,3,4-tetrahydropyrimido[1,2-a]benzimidazole as CRF-1 Receptor Antagonists: Combined QSAR, Glide Docking, Molecular Dynamics, and *In-silico* ADME Studies

Sunil Kumar, Neeraj Kumar, Chandra Shekhar Sharma and Shashank Shekhar Mishra*

*To whom correspondence should be addressed. E-mail: shashankmedchem09@gmail.com

Volume 20, Issue 2 (Spring 2021)

This PDF file includes:
Tables S1-S3

Table S1. 2D structures, observed and predicted values of the compounds according to the best model in 2D and 3D QSAR.

			pIC_{50}				
				2D QSAR		3D QSAR	
**Com. no.**	**Structure**	**IC_{50} (nM)**	**Exp. value**	**Predicted value**	**Residual value**	**Predicted value**	**Residual value**
**B1**	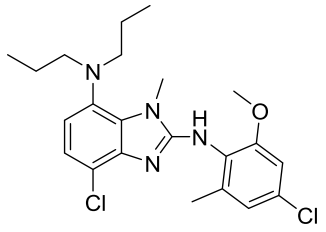	9.5	8.022	8.032	-0.01	7.946	0.076
**B2**	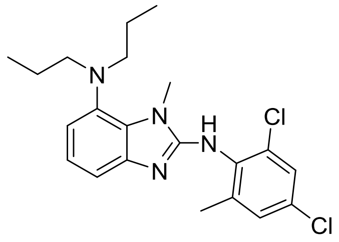	18	7.744	7.799	-0.055	8.085	-0.341
**B3**	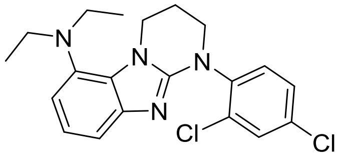	11	7.958	7.721	0.237	7.741	0.217
**B4**	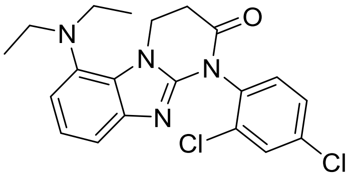	39	7.408	7.621	-0.213	7.231	0.177
**B5**	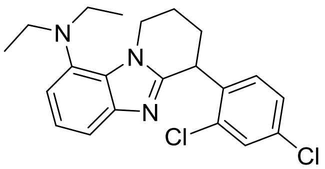	36	7.443	7.721	-0.278	7.309	0.134
**B6**	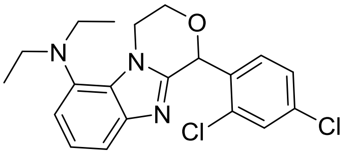	270	6.568	6.673	-0.105	6.979	-0.411

**B7**	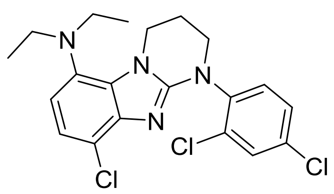	7.1	8.148	7.821	0.327	7.540	0.608
**B8**	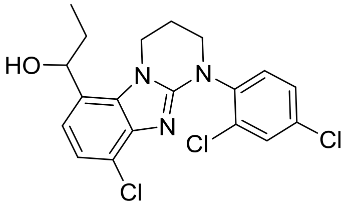	89	7.050	6.842	0.208	7.033	0.017
**B9**	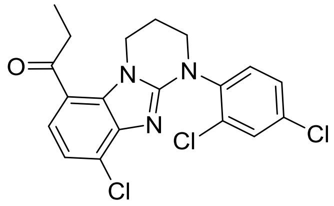	210	6.677	6.642	0.035	6.632	0.045
**B10**	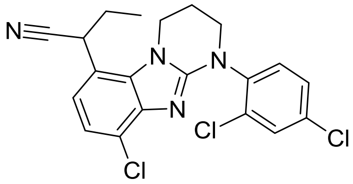	66	7.180	7.421	-0.241	7.164	0.016
**B11**	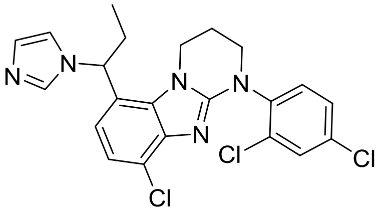	130	6.886	7.19	-0.304	7.312	-0.426
**B12**	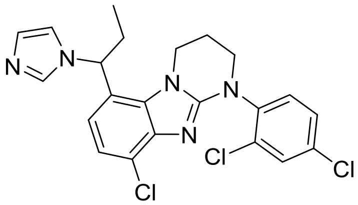	20	7.698	7.196	0.502	7.032	0.666

**B13**	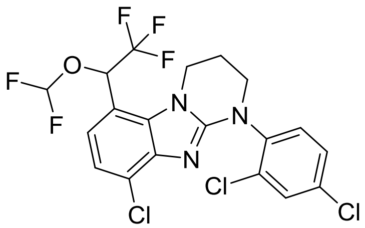	50	7.301	7.349	-0.048	7.328	-0.027
**B14**	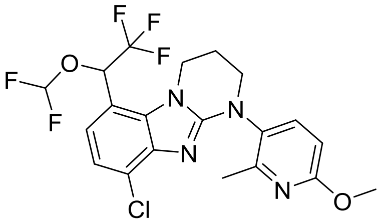	48	7.318	7.080	0.238	7.372	-0.054
**B15**	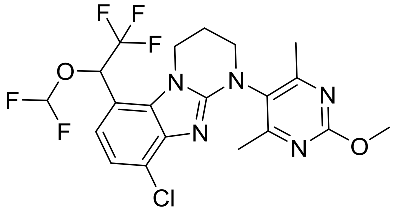	110	6.958	6.780	0.178	6.784	0.174
**B16**	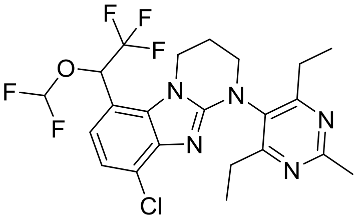	100	7.000	6.912	0.088	6.726	0.274
**B17**	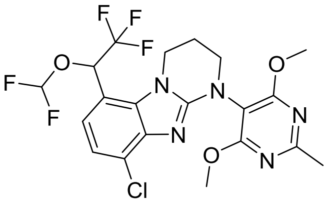	46	7.337	6.912	0.425	7.309	0.028
**B18**	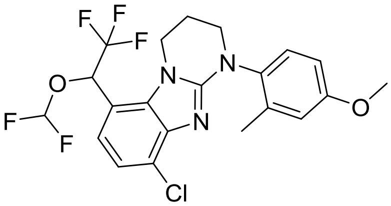	48	7.318	7.480	-0.162	7.269	0.049

**B19**	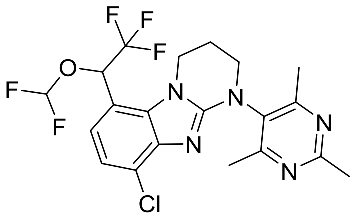	320	6.494	6.648	-0.154	6.979	-0.485
**B20**	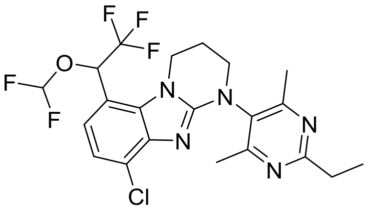	210	6.677	6.780	-0.103	6.740	-0.063

pIC50 (Residual) = pIC50 (Experimental) - pIC50 (Predicted).

Table S2. Calculated relative binding energies of the compounds and reference molecules.

Compound code	dG_Bind	dG_Bind_ Coulomb	dG_Bind_ Covalent	dG_Bind_ Hbond	dG_Bind_ Lipo
**B1**	-57.086	-8.3222	11.3508	-2.318	-48.702
**B2**	-71.049	-3.947	6.7701	-1.261	-40.317
**B3**	-95.794	-9.981	11.293	-0.725	-55.523
**B4**	-98.359	-16.920	7.828	-1.412	-52.789
**B5**	-78.094	-7.439	6.736	-0.704	-41.420
**B6**	-75.203	-6.361	6.761	-0.618	-40.423
**B7**	-107.147	-8.044	6.653	-0.688	-60.386
**B8**	-105.352	-13.870	6.778	-2.030	-56.170
**B9**	-100.907	-13.201	4.377	-1.371	-52.132
**B10**	-101.52	-13.870	9.578	-0.986	-56.173
**B11**	-67.682	-25.223	8.800	-1.200	-42.427
**B12**	-67.682	-25.223	8.800	-1.200	-42.427
**B13**	-100.410	-9.423	8.916	-1.361	-55.225
**B14**	-86.128	-6.256	10.903	-2.024	-48.518
**B15**	-74.371	-7.610	9.027	-1.902	-49.256
**B16**	-49.253	-11.161	17.778	-1.038	-43.773
**B17**	-59.8133	4.539	18.596	-1.432	-46.620
**B18**	-89.375	-6.719	7.090	-1.620	-50.898
**B19**	-77.055	-5.085	8.0784	-2.123	-49.393
**B20**	-78.204	-6.336	10.599	-2.074	-53.344
**Emicerfont**	-62.828	29.558	3.827	-1.308	-29.557
**CP-316,11**	-32.491	-0.161	4.994	-0.125	-39.516
**Verucerfont**	-47.402	1.950	2.869	-2.113	-38.801
**Pexacerfont**	-47.443	-4.047	6.898	-1.015	-38.978

Table S3. Physicochemical parameters calculation results for selected data sets.

Compound code	CNS	Mol.wt.	Volume	QPlogPo/w	QPlogS	QPlogBB	QPPMDCK	Percent Human Oral Absorption
**B1**	1	435.395	1325.782	6.711	-7.343	0.382	10000	100
**B2**	1	405.369	1263.083	6.595	-7.272	0.354	10000	100
**B3**	2	389.327	1165.02	6.096	-6.883	0.581	10000	100
**B4**	1	403.31	1161.242	4.603	-5.436	0.248	9579.757	100
**B5**	2	388.339	1180.042	6.403	-7.243	0.564	10000	100
**B6**	2	390.311	1127.274	5.228	-5.784	0.559	10000	100
**B7**	2	423.772	1207.203	6.603	-7.407	0.803	10000	100
**B8**	1	410.73	1156.253	5.618	-7.15	0.391	10000	100
**B9**	2	408.714	1140.184	5.468	-6.745	0.54	10000	100
**B10**	0	419.74	1219.003	5.916	-8.947	-0.081	8393.701	100
**B11**	2	460.792	1282.862	6.515	-7.886	0.473	10000	100
**B12**	2	460.792	1282.862	6.515	-7.886	0.473	10000	100
**B13**	2	500.682	1219.149	7.901	-9.204	1.335	10000	100
**B14**	2	476.833	1245.375	6.943	-8.198	0.799	10000	100
**B15**	2	491.847	1253.281	6.318	-7.511	0.669	10000	100
**B16**	2	503.902	1357.462	7.07	-8.375	0.723	10000	100
**B17**	2	507.847	1300.24	6.656	-7.79	0.655	10000	100
**B18**	2	475.845	1252.133	7.192	-8.238	0.918	10000	100
**B19**	2	475.848	1262.128	6.399	-7.965	0.75	10000	100
**B20**	2	489.875	1319.843	6.825	-8.37	0.763	10000	100
**Emicerfont**	0	404.471	1282.752	4.985	-7.364	-0.393	777.348	100
**CP-316,11**	1	327.466	1214.753	6.221	-8.527	0.201	5888.187	100
**Verucerfont**	0	412.534	1375.869	4.567	-6.518	-0.322	1619.26	100
**Pexacerfont**	0	340.427	1163.7	4.547	-5.871	-0.066	2402.831	100
**Reference values**[a]	−2 to +2	130.0–725.0	500.0–2000.0	−2 to 6.5	−6.5 to 0.5	−3.0 to 1.2	<25 poor >500great	>80% high <25% poor

[a]Note: range for 95% oral drugs from qikprop.
